# Human gut microbiota analysis of cystic fibrosis infants using metaproteomics

**DOI:** 10.1128/mra.00059-24

**Published:** 2024-07-05

**Authors:** C. García-Durán, C. Saralegui, E. Romeu, M. L. Hernáez, A. Maruri, N. Bastón-Paz, A. Lamas, S. Vicente, E. Perez-Ruiz, I. Delgado, C. Luna-Paredes, J. D. Caballero, J. Zamora, L. Monteoliva, R. Del Campo, C. Gil

**Affiliations:** 1 Dpto. de Microbiología y Parasitología, Universidad Complutense de Madrid and IRYCIS, Madrid, Spain; 2 Servicio de Microbiología, Hospital Universitario Ramón y Cajal and IRYCIS, Madrid, Spain; 3 CIBERINFEC, Madrid, Spain; 4 Unidad de Fibrosis Quística, Hospital Universitario Ramón y Cajal, Madrid, Spain; 5 Unidad de Proteómica, Universidad Complutense de Madrid, Madrid, Spain; 6 Servicio de Pediatría, Hospital Universitario Ramón y Cajal and IRYCIS, Madrid, Spain; 7 Unidad de Fibrosis Quística, Hospital Regional Universitario de Málaga, Málaga, Spain; 8 Unidad de Fibrosis Quística, Hospital Virgen del Rocío, Seville, Spain; 9 Centro de Investigación Biomédica en Red de Enfermedades Respiratorias (CIBERES), Instituto de Salud Carlos III, Madrid, Spain; 10 Universidad de Sevilla, Seville, Spain; 11 Sección de Neumología y Alergia Infantil, Unidad Multidisciplinar Fibrosis Quística, Hospital Doce de Octubre, Madrid, Spain; 12 Unidad de Bioestadística, Hospital Universitario Ramón y Cajal and Instituto Ramón y Cajal de Investigación Sanitaria and Centro de Investigación Biomédica en Red de Epidemiología y Salud Pública (CIBERESP), Madrid, Spain; 13 Universidad Alfonso X El Sabio, Madrid, Spain; University of Notre Dame, Notre Dame, Indiana, USA

**Keywords:** metaproteomics, gut microbiota, cystic fibrosis

## Abstract

We report a metaproteomic analysis of the gut microbiota of eight infants with cystic fibrosis, during the first year of life. This is the first study in this disease that uses metaproteomics to analyze stool samples from patients at such a young age.

## ANNOUNCEMENT

The gut microbiota influences all aspects of our health (1). Previous microbiota studies have focused solely on taxonomy, but current research is focusing on more important functional aspects (2). Few metaproteomics studies have examined gut microbiota in cystic fibrosis (CF), a genetic disease characterized by impaired respiratory and digestive function (3, 4). In the present study, we analyzed the gut microbiota of eight infants diagnosed with CF using samples collected at various time-points, ranging from 1 month to 1 year of life, to investigate the establishment of gut microbiota in CF newborns.

From eight infants at three Spanish hospitals, 30 samples were collected and stored at −80°C until analysis. Table 1 summarizes information regarding the samples. All infants had been diagnosed with CF by neonatal screening, sweat chloride test, and mutation sequencing. Ethical approval was obtained from the ethic committee of Ramón y Cajal Hospital in 2017 (number 163/17), and all parents gave informed consent.

**TABLE 1:** Summary of samples analyzed in this study and metrics of metaproteome analysis

Sample/patient		Time (mo)	Sex	Delivery mode	Birth feeding	CF mutation	Microbial identifications				Human identifications			
							No. peptides		No. protein groups		No. peptides		No. protein groups	
							Per sample	Per patient	Per sample	Per patient	Per sample	Per patient	Per sample	Per patient
1^*a*^	1	1	F	Vaginal	Formula	G542X/ R1162X	1,877	3,926	768	1,522	203	340	57	79
2		2					2,279		950		181		46	
3^*a*^		8					1,159		488		151		51	
4^*a*^	2	1	M	Cesarian section	Formula	F508del/ Y1092X	937	3,613	318	1,421	542	788	136	175
5		5					1,469		628		357		109	
6^*a*^		7					2,133		864		169		69	
7^*a*^	3	1	F	Vaginal	Exc. breast milk^*b*^	F508del/ F508del	1,608	16,966	504	5,432	273	837	56	133
8		4					4,000		1,510		384		87	
9		10					7,007		1,926		180		50	
10^*a*^		12					7,004		2,677		488		85	
11^*a*^	4	1	M	Vaginal	Exc. breast milk^*b*^	F508del/ 3272–26A->G	7,551	19,186	2,001	4,855	416	521	65	84
12		3					6,881		1,943		357		43	
13		6					11,189		3,218		388		49	
14^*a*^		8					8,394		2,432		411		63	
15^*a*^	5	1	M	Vaginal	Exc. breast milk^*b*^	F508del/ F508del	2,595	6,287	738	1,943	148	600	38	83
16		4					2,535		779		535		67	
17		6					2,246		881		187		48	
18^*a*^		8					779		332		89		29	
19^*a*^	6	1	M	Vaginal	Formula	F508del/ N1303K	3,779	19,582	1,273	5,837	301	1,053	70	125
20		2					8,036		2,405		281		54	
21		6					8,133		2,615		448		78	
22^*a*^		9					4,532		1,806		604		81	
23^*a*^	7	1	F	Vaginal	Formula	F508del/ F508del	2,544	6,374	449	2,017	863	1,375	132	252
24		4					2,354		666		533		120	
25		5					2,142		635		342		104	
26^*a*^		7					2,743		745		551		117	
27^*a*^	8	1	F	Vaginal	Formula	F508del/ R1162X	4,915	11,530	979	3,840	496	1,438	108	250
28		3					4,924		639		1,077		149	
29		5					3,187		1,093		485		140	
30^*a*^		11					3,487		1,073		539		119	

^*a*^ Samples that were used for gut microbiota analysis to assess differences between initial (1-month samples) and early CF samples or final samples (7- to 12-month samples).

^*b*^ Exclusively breast milk. All infants included solid food at endpoint.

Samples were processed following a previously published protocol (5). Briefly, a 0.1–0.3 g feces sample was suspended in 10 mL phosphate buffer and mixed for 45 min at 4°C in a tube rotator. Samples were centrifuged for 5 min, 500 *g*. This process was repeated twice, and then the three supernatants were centrifuged at 11,000 *g*, 20 min. Microbial pellets were suspended in 500 mL lysis buffer (4% sodium dodecyl sulfate; 50 mM Tris-HCl, pH 8.0) and heated for 10 min at 95°C. Samples were subjected to four sonication cycles (30 s, amplitude of 40%) and five rounds of bead-beating (30 s, speed of 6.5 ms^–1^), followed by centrifugation at 14,000 *g*. Proteins were precipitated using methanol-chloroform and suspended in 8 M urea. After quantification with Qubit 3.0 (Thermo Fisher Scientific), 50 µg of protein were reduced using 10 mM dithiothreitol (45 min, 37°C) and alkylated with 55 mM iodoacetamide (30 min, 25°C), followed by digestion with 1:25 trypsin (enzyme:protein; ≥150 U/mg) (37°C, overnight). Next, the peptides were desalted and concentrated with C_18_ reverse-phase chromatography (OMIX C_18_; Agilent technologies) and then resuspended in 12 µL 2% acetonitrile/0.1% formic acid. Finally, 1 µg of peptides was loaded for mass spectrometric analysis on an EASY-nLC 1000 system (Thermo Fisher Scientific) coupled to a Q-Exactive HF mass spectrometer (Thermo Scientific). Peptides were loaded onto an Acclaim PepMap 100 trapping column (Thermo Scientific) and then separated on a C_18_ resin analytical column (Thermo Scientific) using a 240 min gradient from 2% to 40% buffer B (0.1% formic acid in 100% acetonitrile) in buffer A (0.1% formic acid). Data were obtained by data-dependent acquisition in positive mode, using Xcalibur 4.1 software. From each MS scan (350–2,000 Da), the 15 most intense precursors (charges 2–4) were selected for their high collision energy dissociation fragmentation (dynamic exclusion of 10 s and normalized collision energy of 27).

Data were processed using MetaLab software (version 2.0) (6), which provides the Integrated Gene Catalog with 9,878,647 sequences (https://db.cngb.org/microbiome/genecatalog/genecatalog_human/) (6, 7). For the identification of human proteins, we also used a database downloaded from UniProt DB (8), restricted to human taxonomy (downloaded on 18 February 2020 with 74,451 sequences). MaxQuant (version 1.6.9.0) was employed for peptide identification and quantitative analysis (using maxLFQ algorithm). Default parameters were used, except where otherwise noted. Search parameters included carbamidomethylation of cysteines as fixed modification, oxidation of methionine and N-terminal acetylation as variable modifications, and a maximum of 2 missed cleavages allowed. For peptide and protein identification, the FDR was set to 0.01. For our analysis, we only considered proteins having at least one unique peptide, and taxa identified with at least two peptides. For taxonomic analysis, the intensities of all of the distinctive peptides assigned to a taxon were summed to determine the relative abundance of that taxon, excluding human peptides. Data analysis and visualization were performed with phyloseq package (version 1.42.0) (9) of RStudio (version 4.2.2). The code used in this announcement can be found in github (https://github.com/Carmengar/Metaproteomic_gut-microbiota_cystic_fibrosis).

**Fig 1:**
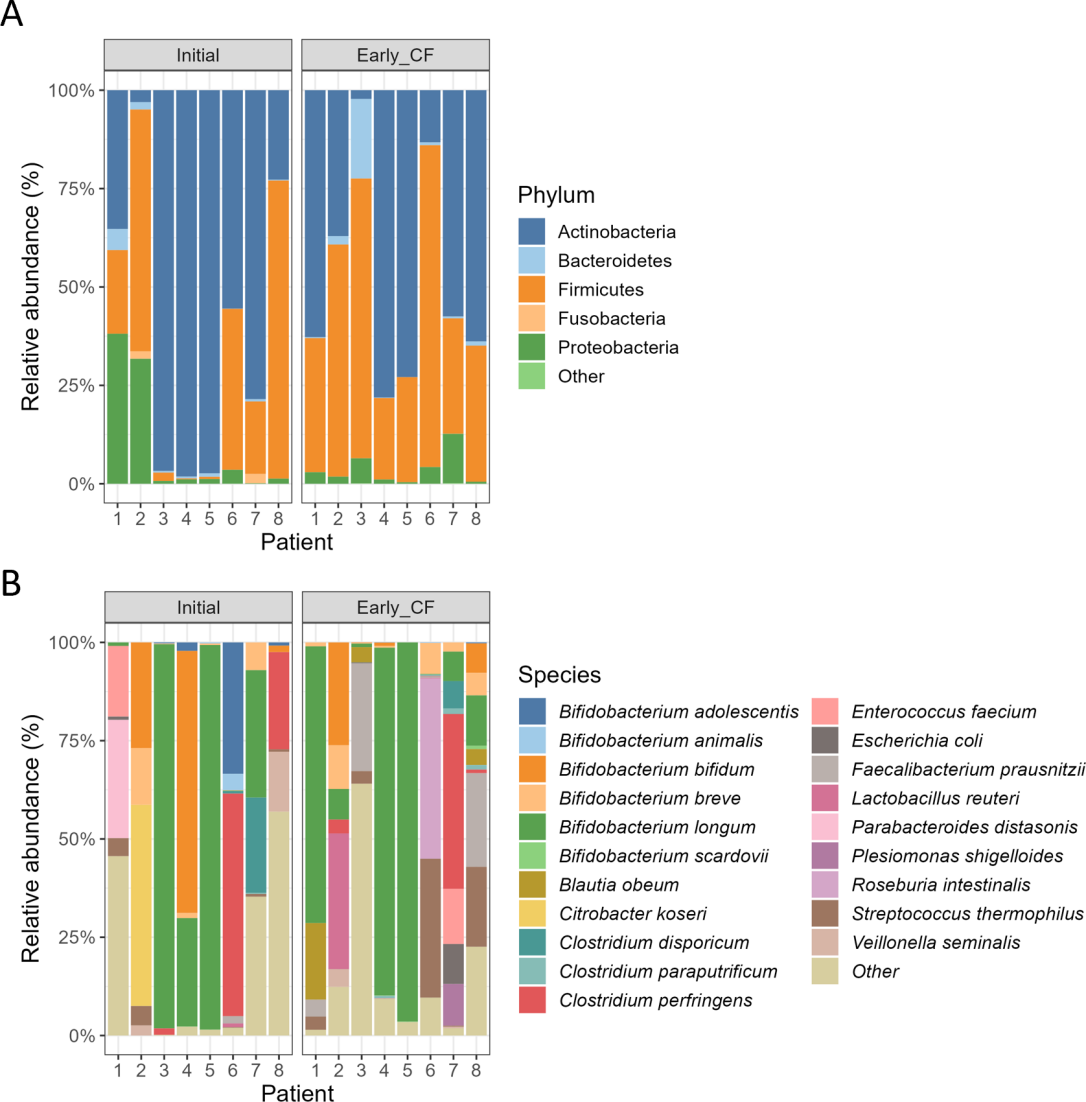
Taxonomic composition of gut microbiota of CF infants based on metaproteomic analysis. The relative abundance of each taxon is represented at (**A**) phylum and (**B**) species level. At thephylum level, only taxa representing more than 1% of abundance is present, while at the species level, only the 20 top most abundant taxa are represented. Less abundant taxa are represented at the “Other” category in both taxonomic levels.

We identified and quantified a total of 24,170 protein groups and 62,120 peptide sequences, corresponding to 300 taxa, including 67 genera and 133 species. The microbial composition was characterized by an abundance of phylum Actinobacteria in the initial samples, and an increase of phylum Firmicutes in the early CF samples (Fig. 1). We also identified 293 human proteins (Table 1), which could be of great interest for the study of host-pathogen interactions. In conclusion, this data set could be very useful for scientists working in the field of cystic fibrosis. The metaproteomic results of these samples were further analyzed in Saralegui et al. (10).

## Data Availability

The raw MS proteomics data have been submitted to the ProteomeXchange Consortium (http://www.proteomexchange.org) via the Proteomics Identifications Database (PRIDE) partner repository, with the database identifier PXD029284 (11).
